# Obstructive sleep apnea syndrome (OSAS) in mouth breathing children

**DOI:** 10.1590/S1808-86942010000500003

**Published:** 2015-10-22

**Authors:** Suemy Cioffi Izu, Caroline Harumi Itamoto, Márcia Pradella-Hallinan, Gilberto Ulson Pizarro, Sérgio Tufik, Shirley Pignatari, Reginaldo Raimundo Fujita

**Affiliations:** 1 MD. ENT Resident physician - Federal University of São Paulo - Medical School (UNIFESP-EPM); 2 MD. ENT Resident physician - UNIFESP-EPM; 3 PhD - UNIFESP-EPM, Physician at the Pediatrics Polysomnography Ward - Department of Psychobiology - UNIFESP- EPM; 4 MSc in Otorhinolaryngology - UNIFESP- EPM, ENT Physician; 5 Associate Professor of Psychobiology - UNIFESP-EPM, Full Professor and Head of the Psychobiology Department of the UNIFESP-EPM; 6 Post-Doctorate degree in ENT - UNIFESP-EPM, Adjunct Professor and Head of the Pediatric Otorhinolaryngology Program; 7 PhD in Otorhinolaryngology - UNIFESP-EPM, Adjunct Professor and Head of the Pediatric Otorhinolaryngology Program - UNIFESP - EPM. Universidade Federal de São Paulo - Escola Paulista de Medicina

**Keywords:** sleep apnea, children, polysomnography, prevalence, mouth breathing, snoring.

## Abstract

**Abstract:**

It is well known that mouth breathing is associated with adenotonsillar hypertrophy - which is the main cause of obstructive sleep apnea among children. Despite the importance of this matter, there are only a handful of studies showing the relationship between OSAS and mouth breathing.

**Aim:**

to determine the prevalence of obstructive sleep disorders in mouth breathing children and study its correlation with otorhinolaryngological findings.

**Study design:**

Retrospective cohort study.

**Method:**

Data analysis from 248 medical charts of mouth breathing children seen at the Pediatric Otolaryngologic Division of a large medical institution between the years of 2000 and 2006. All patients had nasofibroscopy and or Cavum radiographs and polysomnographic exams. According to the Apnea index, patients were classified as primary snorers (AI<1); and as OSAS (≥1).

**Results:**

From 248 patients included in the study, 144 (58%) were primary snorers and 104 (42%) had OSAS. The most prevalent otorhinolaryngological findings were adenotonsillar hypertrophy (n=152; 61.2%), tonsilar hypertrophy (n=17; 6.8%), adenoid hypertrophy (n=37; 14.9%), rhinitis (n=155; 62.5%) and secretory otitis (n=36; 14.5%).

**Conclusions:**

primary snoring and OSAS are frequent findings in mouth breathing children. The most frequent otorhinolaryngological disorder in children with OSAS is adenotonsillar hypertrophy with or without rhinitis.

## INTRODUCTION

Snoring and oral breathing are frequent complaints which make the parents of children with these symptoms to take them to otorhinolaryngologists. The prevalence of habitual snoring in children between 3 and 13 years varies between 5.2 and 34.45%^1^, ^2^, ^3^, ^4^, while the prevalence of oral breathing is of 26.8% according to a large study with 661 children aged between 6 and 12 years^5^.

Chronic mouth breathing in children is usually associated with palatine and pharyngeal tonsils hypertrophy,^4^ with or without allergic rhinitis. Its incidence peak happens in pre-school aged children^6^,^7^. At this stage, besides the natural growth of the soft palate and pharyngeal tonsils, there are repetition infections which cause tonsillar lymphoid tissue hypertrophy, changing the breathing of children to a chronic obstructive pattern. A consequence of this in the long run has been craniofacial changes^8^ which maintain the mouth breathing pattern, besides causing postural^9^ and hearing^10^ disorders. Among mouth breathers, it is also common to find the Obstructive Sleep Apnea Syndrome (OSAS)^11^,^12^, a potentially severe clinical situation, as well as primary snoring.

Primary snoring is noise produced by breathing, caused when air passes through the upper airway without, however, causing changes to sleep, alveolar ventilation and oxygenated hemoglobin saturation. It is common during childhood, happening from 7 to 10% of the children between 1 and 10 years^17^. Obstructive Sleep Apnea Syndrome (OSAS) in children is a disease characterized by partial prolonged and/or complete obstruction of the upper airways, impairing normal ventilation. The signs and symptoms of this syndrome include the common snoring, interrupted sleep, neurocognitive and behavioral disorders such as learning disorders^13^,^14^, behavioral changes, attention deficit and hyperactivity^13^,^14^. The major complications of the OSAS include growth and development delays^15^, mental retardation^15^ and cor pulmonale^16^. The gold standard test used to diagnose OSAS is Polysomnography^18^, since it enables us to differentiate OSAS from primary snoring^19^. Its high cost and difficulty in performance in children are, however, important limitations and also reasons to explain why only a few centers can count on this exam for most of its patients.

Given the importance of OSAS in children and the scarcity of studies correlating this disease to mouth breathing in children, we designed this study with the goal of determining OSAS prevalence in mouth breathing children and check its correlation with otorhinolaryngological findings.

## METHOD

The data was obtained from the charts of patients who visited the pediatric otorhinolaryngology ward of a teaching hospital between 2000 and 2006. In the study we included children with ages ranging between 0 and 13 years, diagnosed by an multidisciplinary team (otorhinolaryngologists, allergists, speech and hearing therapists, orthodontists, physical therapists) as mouth breathers, using the criteria of preferentially oral breathing for more than 6 months and physical exam showing two or more signs of oral breathing (nasal obstruction, deep hard palate, maxillary atresia, open mouth, mouth muscle laxity, bite changes, posture changes and anterior flexion of the head).

Children diagnosed as mouth breathers were submitted to sleep disorder investigation by means of nocturnal polysomnography under the following protocol: in a dark and silent room and with the child's guardian present. The electrophysiological and cardio-respiratory parameters were recorded in a computerized system (Alice 3 Healthdyne/respironics, Marrieta, GA), using EEG data ( C3/A2, C4/A2, O1/A2, O2A1), submentonian and tibial electromyogram, right and left side electro-oculogram, oronasal airflow, chest and abdominal movement, laryngeal microphone (snoring), oxyhemoglobin saturation (SaO2) and position on the bed. The test was analyzed by a physician with experience with the pediatric population and it is classified according to criteria from the American Thoracic Society (1995).

We excluded those children with genetic syndromes, metabolic disorders, neurologic diseases or congenital malformations.

From each chart we analyzed the following information: gender, age at the time of the polysomnography, ENT diagnosis and polysomnographic diagnosis, hypopnea/apnea ratio (HAR) and the rate of oxygen saturation (satO_2_).

According to the “American Thoracic Society”^19^, we consider the following:

– Hypopnea/apnea index (HAI): number of obstructive and mixed apnea episodes in a minimum time span of two respiratory cycles (expressed in episodes/hour). OSAS is diagnosed in children with AI >1 / hour.
– O_2_ Saturation Nadir: minimum oxygen saturation during the sleep study. Values below 90% are associated with central or obstructive sleep ventilatory disorders.

The otorhinolaryngological diagnoses of each child were based on the initial otorhinolaryngological evaluation, including nasal-fibroscopy.

The data was grouped according to the results from polysomnography tests and all classifications were based on the HAI and O_2_ Saturation Nadir values.

The individuals were broken down into primary snorers when they had HAI < 1, and those with OSAS, when the AI was ≥ 1. The children with OSAS were further divided into mild (1 ≤ HAI< 5), moderate (5 ≤ HAI < 10) and severe (HAI ≥ 10)^20^ OSAS. Within each subgroup, there was also one more division, according to the degree of O_2_ desaturation, considering the nadir (minimum level of O2 saturation). Thus, we considered four subgroups: Nadir = 80; 80 < Nadir = 85; 85 < Nadir =90 e Nadir = 90. The data obtained was associated with patient age and gender and the findings from the evaluation and otorhinolaryngological exams.

The patients signed the free and informed consent form approved by the Ethics in Research Committee of the Federal University of São Paulo Medical School - Unifesp-EPM, protocol 171/06 approved on February 10, 2006.

For the statistical analysis we used the Chi-squared^28^ test.

## RESULTS

**Chart 1:**
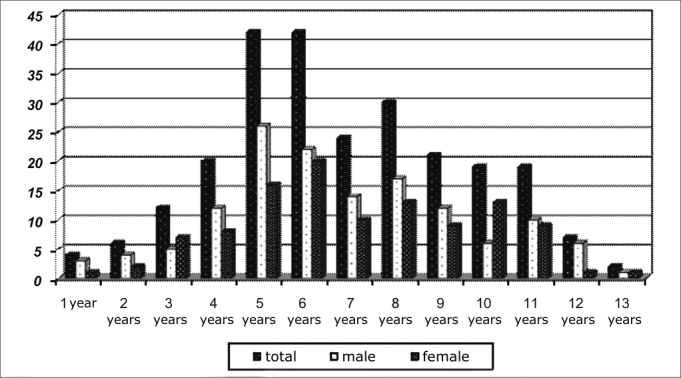
Distribution of sleep respiratory disorders according to gender and age.

The final sample comprised 248 patients. Among them, 144 (58%) had primary snoring and 104 (42%) had OSAS. The peak occurrence of sleep disorder happened between 4 and 7 years. The prevalence of OSAS among males was 60% (62) and among females was 42% (42). Chart 1 shows the distribution of sleep respiratory disorders according to gender in the different age ranges.

**Chart 2:**
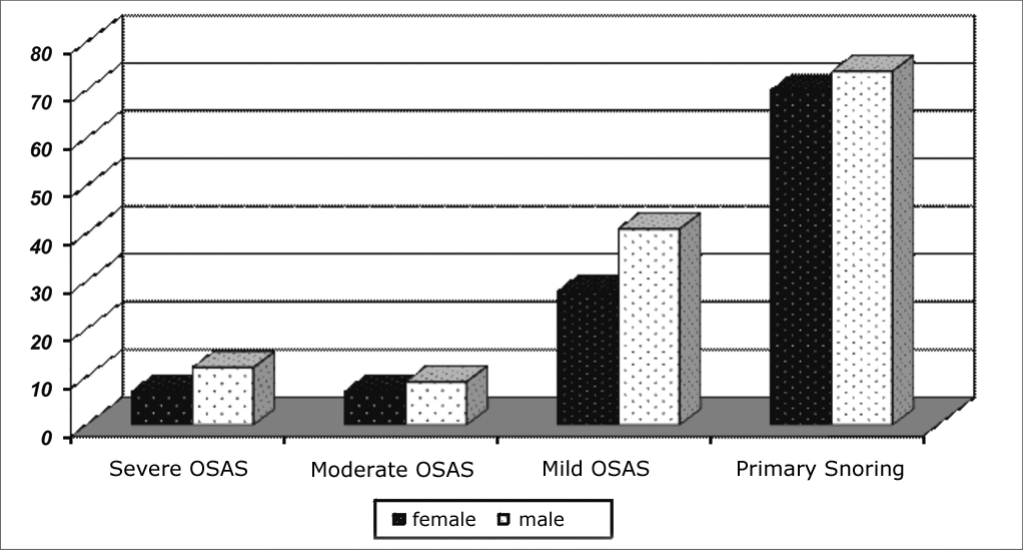
Distribution of sleep respiratory disorders according to age and gender.

Among the 104 patients with OSAS, 69.67% had the mild type; 16.15%, had the moderate type and 19.18% had severe OSAS. Chart 2 shows that the same ratios were obtained when we break down respiratory sleep disorders according to gender.

**Chart 3:**
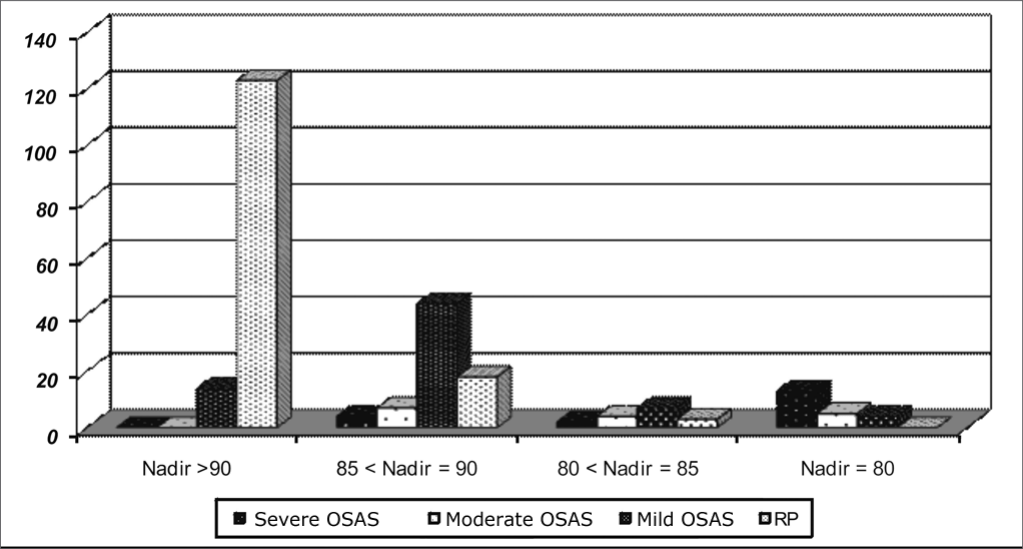
O_2_ saturation Nadir in sleep respiratory disorder.

The level of O_2_ desaturation varied as depicted on Chart 3. The lower nadir values were found among severe and moderate OSAS.

**Chart 4:**
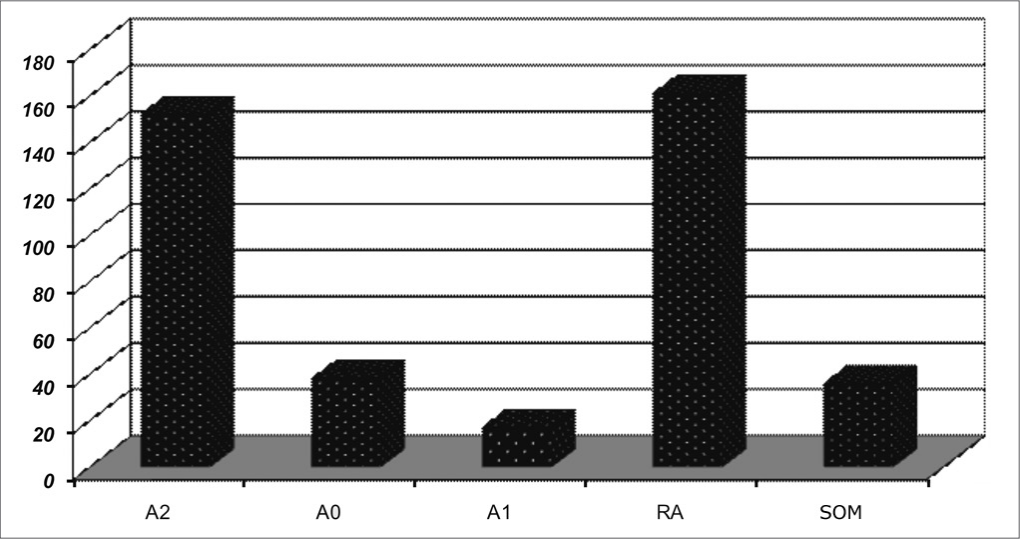
ENT findings in patients with sleep respiratory disorders.

**Chart 5:**
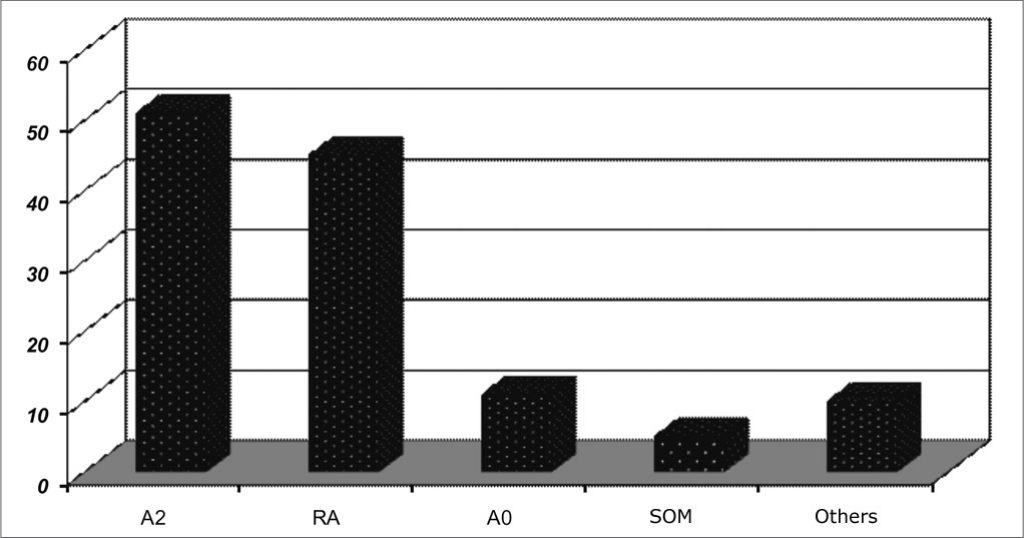
ENT findings in patients with mild OSAS.

**Chart 6:**
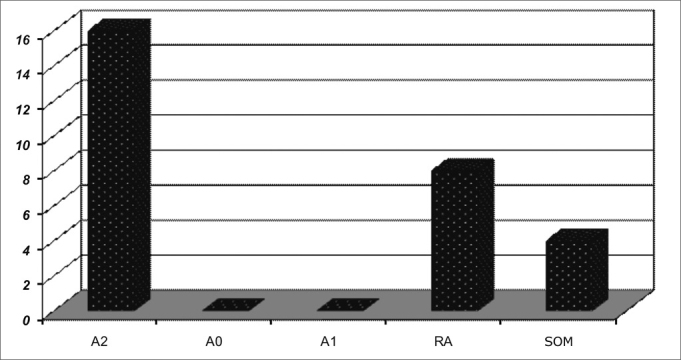
ENT findings in patients with moderate OSAS.

**Chart 7:**
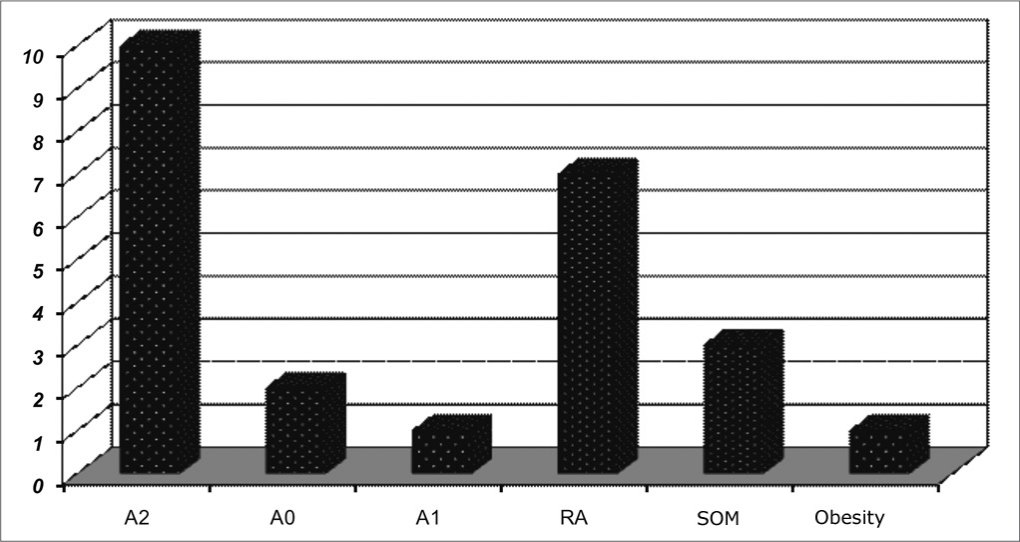
Clinical and ENT findings n patients with severe OSAS.

Otorhinolaryngological findings are listed on Chart 4, Chart 5, Chart 6, Chart 7, which show the distributions of the most commonly found Otorhinolaryngological disorders, according to the types and levels of sleep respiratory disorders.

In general, the most frequently found ENT alterations are: adenoid and tonsil hypertrophy (n=152; 61.2%), palatine tonsil hypertrophy (n=17; 6.8%); pharyngeal tonsil hypertrophy (n=37; 14.9%), allergic rhinitis (155; 62.5%) and secretory otitis media (36; 14.5%).

In all groups, adenoid and tonsil hypertrophy associated with allergic rhinitis were the most common finding.

The correlation between mouth breathing and OSAS was significant for adenoid and tonsil hypertrophy (p=0.001), and there were no significant differences concerning children with allergic rhinitis, tonsil hypertrophy, adenoid hypertrophy and nasal septum deviation.

## DISCUSSION

Despite the huge attention the medical community has been paying to sleep respiratory disorders lately, the literature is still relatively poor as far as the pediatric population is concerned.

Our study was carried out with mouth breathing children whom, based on epidemiological studies, can vary between 20 and 40%7,8 of the general pediatric population. We have noticed that the frequency of these disorders, especially primary snoring and OSAS, is very significant in this particular population.

In this study, the prevalence of OSAS was higher among boys, which was not reported by prior epidemiological studies^7^,^21^, ^22^, ^23^, ^24^. Nonetheless, in the adult population, males are more often affected^25^,^26^. This happens because of the influence of male sex hormones in respiratory control and/or body fat distribution.

We should bear in mind that our institution has a huge backlog of patients waiting for adenotonsillar surgery (as it happens in other large institutions). In some cases, the patients wait for more than three years. It is likely that mothers, concerned with their daughters (female snoring is more disruptive than in males) went to other institutions, and for that reason we lost follow up in our ward.

The peak of prevalence in both genders happened between 4 and 7 years, an age in which adenoid and the tonsils naturally grow. Moreover, it is possible that with children going to day-care centers and schools, a factor known to predispose children to repetitious upper airway infection, may also represent a worsening factor in the induction of adenotonsillar hypertrophy, worsening even further the respiratory condition of the children in this age range.

Although moderate and severe OSAS have been less frequent in relation to other sleep disorders, they were present in 35 (14%) children and, as expected, these were the ones who had the highest saturation drops. It is also interesting to stress that it was only in the group of severe OSAS that children were obese, although obesity is not an associated factor. This data matches sleep studies in obese children^27^.

The most commonly found otorhinolaryngological disorder in patients with sleep respiratory disorder was palatine and pharyngeal tonsil hypertrophy, either with or without allergic rhinitis. Such finding coincides with data from prior studies published in the literature on OSAS etiology pointing to adenoid and tonsil hypertrophy as the main causes of OSAS in children^3^,^5^. Chronic secretory otitis media (SOM), was also a very frequent ENT disorder found in our patients. This fact is probably associated with mouth breathing^10^ and the presence of pharyngeal tonsil hypertrophy, a major cause of mouth breathing and Eustachian Tube dysfunction in children.

The results from our study show the importance of sleep respiratory disorders in the pediatric population by its frequency. Nonetheless, it is important to stress the major clinical relevance of such disorders which, as shown in prior studies^6^,^7^,^13^, ^14^, ^15^, ^16^, made the children more prone to hyperactivity and attention deficit, besides impairing learning. Therefore, in regards of the child's development, the knowledge about the real incidence of apnea in the pediatric population complaining of mouth breathing is very important, because of the need for an early intervention which can prevent carriers from developing school problems and, consequently, social and psychological ones. Since the otolaryngologist and the pediatrician are the first to have contact with this type of patient, it is very important that these professionals have an eye open for the diagnosis of sleep disorders, thus making the intervention early and correct.

The follow up of these patients after clinical or surgical treatment of the associated disorders, including polysomnography, may contribute to the knowledge of the natural history of these disorders and its physiopathology.

## CONCLUSIONS

1 The incidence of OSAS occurrence in the mouth breathing children of our study was 42%.
2 In these children, the OSAS prevalence peak happened between 4 and 7 years of age.
3 The most frequently found ENT disorder in OSAS children was adenoid and tonsil hypertrophy with or without allergic rhinitis.

## ACKNOWLEDGEMENTS

AFIP
